# Pattern of improvement in upper limb pointing task kinematics after a 3-month training program with robotic assistance in stroke

**DOI:** 10.1186/s12984-017-0315-1

**Published:** 2017-10-13

**Authors:** Ophélie Pila, Christophe Duret, François-Xavier Laborne, Jean-Michel Gracies, Nicolas Bayle, Emilie Hutin

**Affiliations:** 1 Centre de Rééducation Fonctionnelle Les Trois Soleils, Médecine Physique et de Réadaptation, Unité de Neurorééducation, 19 rue du Château, Boissise-Le-Roi, 77310 France; 2 EA 7377 BIOTN, Laboratoire Analyse et Restauration du Mouvement (ARM), Université Paris-Est Créteil, Hôpitaux Universitaires Henri Mondor, Assistance Publique - Hôpitaux de Paris, 51 Avenue du Maréchal de Lattre de Tassigny, Créteil, 94010 France; 3 SAMU 91, Centre Hospitalier Sud Francilien, 116 Boulevard Jean Jaurès, Corbeil-Essonnes, 91100 France

**Keywords:** Hemiparesis, Subacute stroke, Prolonged robot-assisted training, High intensity, Repetitive active movements

## Abstract

**Background:**

When exploring changes in upper limb kinematics and motor impairment associated with motor recovery in subacute post stroke during intensive therapies involving robot-assisted training, it is not known whether trained joints improve before non-trained joints and whether target reaching capacity improves before movement accuracy.

**Methods:**

Twenty-two subacute stroke patients (mean delay post-stroke at program onset 63 ± 29 days, M2) underwent 50 ± 17 (mean ± SD) 45-min sessions of robot-assisted (InMotion™) shoulder/elbow training over 3 months, in addition to conventional occupational therapy. Monthly evaluations (M2 to M5) included Fugl-Meyer Assessment (FM), with subscores per joint, and four robot-based kinematic measures: mean target distance covered, mean velocity, direction accuracy (inverse of root mean square error from straight line) and movement smoothness (inverse of mean number of zero-crossings in the velocity profile). We assessed delays to reach statistically significant improvement for each outcome measure.

**Results:**

At M5, all clinical and kinematic parameters had markedly improved: Fugl-Meyer, +65% (median); distance covered, +87%; mean velocity, +101%; accuracy, +134%; and smoothness, +96%. Delays to reach statistical significance were M3 for the shoulder/elbow Fugl-Meyer subscore (+43%), M4 for the hand (+80%) and M5 for the wrist (+133%) subscores. For kinematic parameters, delays to significant improvements were M3 for distance (+68%), velocity (+65%) and smoothness (+50%), and M5 for accuracy (+134%).

**Conclusions:**

An intensive rehabilitation program combining robot-assisted shoulder/elbow training and conventional occupational therapy was associated with improvement in shoulder and elbow movements first, which suggests focal behavior-related brain plasticity. Findings also suggested that recovery of movement quantity related parameters (range of motion, velocity and smoothness) might precede that of movement quality (accuracy).

**Trial registration:**

EudraCT 2016–005121-36. Date of Registration: 2016–12-20. Date of enrolment of the first participant to the trial: 2009–11-24 (retrospective data).

## Background

Following stroke, 70 to 90% of patients report residual motor impairment in their paretic upper limb, affecting daily activities and quality of life [1–5]. The recovery of motor function results in part from neural re-organization, which is facilitated by early onset of rehabilitation care [6] and high intensity of training programs [7, 8]. High intensity may relate to extended program durations, increased frequencies of rehabilitation sessions or to an increased number of specific movements or tasks achieved per session [9–11].

The use of robotic devices in spastic paresis helps deliver high dosages of physical treatment, based on high number repetition of goal-directed tasks in an interactive environment [12–17]. A number of controlled clinical trials have suggested positive effects of robot-assisted training programs, applied in complete or partial substitution of or in adjunction to conventional occupational therapy, on upper limb function in subacute and chronic stroke [12–17]. Overall, greater motor improvements were reported with robot-assisted training programs when compared with conventional therapy programs [12–16], except when a matched intensity level of exercise was used in manual therapy, which is unusual or difficult in conventional rehabilitation [17–19]. In addition to potentially enhancing motor improvement after stroke, robotic devices comprise goniometers and sensors of position, force and stiffness, and thus can provide immediate, reliable and continuous measurements of the movements performed during the training sessions [20–28]. In contrast to clinical scales, these robot-based kinematic assessments might shed some insight on the mechanisms of motor recovery that occur after stroke, and provide the clinicians with useful information that could help them adjust the components and schedule of physical treatments [28]. Although, upper limb motor improvements based on Fugl-Meyer Assessment are well documented over the subacute phase [29–31], longitudinal and comprehensive explorations of the relationships between the improvements of clinical scores and of robot-based kinematic assessments in the late subacute phase are still scarce [24, 25]. Moreover, to our knowledge, prior studies did not use intensive and highly repetitive programs including robot-assisted training delivered over a prolonged period of time in the subacute stroke population. On the other hand, precise use of clinical scales might help understand the site-specificity of the training-induced recovery. While preferential improvement in specifically trained body parts has been reported between the upper and lower body [7], site-specific improvements within a limb are less well documented. When considering lesion-induced - not behavior-induced - brain plasticity, some studies have suggested greater difficulties in generating forces from distal versus proximal limb segments, a finding that remains controversial [32, 33]. With respect to behavior-induced plasticity in stroke, there is conflicting evidence of how focal effects of training may be within a paretic limb [14, 15, 34–36]. To further justify, or dispute, the validity of ongoing investment into robot-based rehabilitation technologies, a refinement of our knowledge on robot-induced effects is required.

The two main objectives of the present study were to measure the overall changes associated with a 3-month robot-assisted training program coupled with conventional care, on motor impairment and pointing task kinematics of the upper limb in late subacute stroke (from late 2nd to late 5th month post stroke, a time period infrequently explored), and to compare the course of the various kinematic parameters over time, and the associated clinical changes at different joints.

## Methods

### Subjects

This retrospective study was conducted in the Neurorehabilitation Department at “Les Trois Soleils” Center, Boisisse-Le-Roi, France, in accordance with the Declaration of Helsinki (2008), Good Clinical Practice guidelines and local regulatory requirements. This study was approved by the local Committee for the Protection of Persons (CPP Ile de France 1). All patients gave an informed consent before inclusion in the study. Patient charts were reviewed based on the following inclusion criteria: age over 18, single, first stroke event confirmed on CT (computerized tomography) or MRI (Magnetic Resonance Imaging), completion of a 3-month robot-assisted training program for the paretic shoulder and elbow during the sub-acute phase after stroke, a Fugl Meyer score under 35 at the onset of the rehabilitation program, and participation in monthly clinical and robot-mediated assessments from late 2nd to late 5th month after stroke. In addition, 17 healthy subjects (Age, 53 ± 18, 9 female) without known neurological or orthopedic disorders participated to generate control kinematic data (see evaluation procedures).

### Robot device

**Fig. 1:**
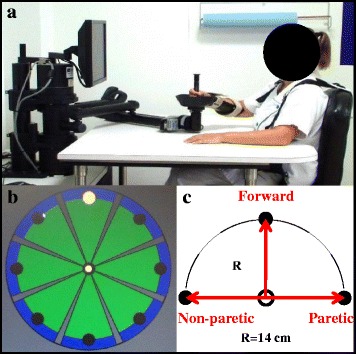
InMotion 2.0 shoulder/elbow robotic system. **a** Therapist using the InMotion 2.0 shoulder/elbow robotic system; **b** Pointing task interface; **c** Pointing tasks used in the kinematic analysis. Only the three directions analyzed for the study are represented; paretic and non-paretic directions are indicated here for a patient with right hemiparesis

We used an end-effector robotic system equipped with 2 translational degrees of freedom emphasizing shoulder and elbow movements from supported hand displacement in the horizontal plan (InMotion 2, Interactive Motion Technologies, Inc., Watertown, MA, Fig. 1a) [36]. The robot provides continuous assistance-as-needed to movement using an adaptive algorithm [37].

### Physical treatment

All patients underwent a rehabilitation program focused on the upper limb, which combined robot-assisted therapy with conventional occupational therapy, each for 45 min per day, 5 days per week. During the robot-assisted therapy sessions, patients were seated in front the screen, with their trunk constrained (strapped using a four-point seatbelt), holding the manipulandum with their affected hand, the forearm supported in a platform. For 30 min of the 45-min robot session, the main tasks were pointing tasks in which patients performed as many repetitive reaching movements as they were able, toward targets in 8 directions, in a clockwise order (not randomized). Each completed movement represented a 14-cm horizontal displacement, the hand of the patient being supported by the arm-plate of the robot (Fig. 1b). In addition to the visual feedback provided by the screen, the therapist (physical or occupational therapist) guiding the patient in the point-to-point tasks kept verbally motivating the patient to achieve the best possible performance. For the remaining 15 min of the robot session, patients practiced other types of reaching tasks. Occupational therapy sessions coupled passive muscle stretching techniques, performed by the clinician, with active reaching movements and specific grasp and release tasks, performed by the patient. Time of training in the day (morning or afternoon) varied according to department schedule and patients.

### Evaluation procedures

Participants underwent four monthly clinical and robot-mediated evaluations, starting two months after the occurrence of the stroke. At each visit, patients were evaluated using the Fugl-Meyer Assessment scale for the upper extremity (FM, [38–40]). We used proximal and distal indices (PXI and DSI), respectively defined as the percentage of the FM shoulder/elbow subscore over the 36 maximum possible points for these joints and the percentage of FM wrist and hand subscores over the maximum possible 24 points there.

In both patients and healthy subjects, the robot-based assessment offered by the evaluation program involved 40 back and forth movements *without* assistance to movement (robot unpowered), five in each of the eight directions requested by the experimenter (overall 80 movements). Robot-derived measurements were then normalized to control data for three of the eight hand trajectories practiced: going forward, towards the paretic and non-paretic sides as therapists have notified these 3 directions as being the most difficult to achieve by patients with hemiparesis in clinical routine (Fig. 1c). To simplify the analysis, only these three directions, classically considered the most difficult for the paretic upper limb (paretic, non-paretic, forward) were thus taken into account to compute the kinematic measures in the present study. For each of these three trajectories, four kinematic measurements were computed:

- the Distance Index (DI) was defined as the mean distance traveled by the subject’s hand from the starting position, in percent of control values, i.e. the means of the values in healthy subjects: a maximum score of 100% indicated that the participant could reach the target (with the arm supported in the robotic device) or even pass it (hypermetria), a rare occurrence in subjects with hemiparesis. Thus, any movement exceeding the required distance was still measured as 100% in terms of Distance Index, as any excessive distance covered (hypermetria) is not counted with the InMotion™ robot.
- the Velocity Index (VE) was the hand velocity (distance traveled divided by movement time) in percent of control values;
- the Accuracy Index (AC) was the inverse of the root mean square error from straight line, in percent of control values; in other words, we computed the area under the curve of the errors between the actual trajectory of the patient’s hand and an ideal direct, linear trajectory from start to target.
- the Smoothness Index (SM) was defined in the present study as the inverse of the mean number of zero-crossings in the velocity profile, in percent of control values [41–43]. Although there are several ways in which one may compute movement smoothness, this method, while it may be less sensitive than other methods in subjects with mild movement impairment, has been used to analyze the number of discrete (sub)movements in severely affected subjects, like the early post stroke subjects of the present study [43]. When motor recovery occurs, the velocity profile of the hand movement presents fewer peaks, resulting in a smoother movement [43]. As a potentially more sensitive metric of smoothness, we also analyzed the inverse of the mean number of zero-crossings in the *acceleration* profile, to verify whether patterns of changes would be similar or not between the two metrics.

### Statistics

To analyze the treatment effects on the clinical scores and kinematic parameters over the four assessment visits (M2, M3, M4, M5), we used a repeated measures analysis of variance (ANOVA), with Bonferroni corrections to adjust for multiple comparisons, except for non-parametric variables (smoothness index, a discrete variable for which we used the Friedman test). Two-way ANOVAs were carried out to explore interactions between time and joint location - proximal (shoulder, elbow) vs distal (wrist, hand) - as potential predictors of the changes in Fugl-Meyer scores and to test time* direction effects on kinematic performance. A *p* value of 0.05 was used for statistical significance.

## Results

Between October 2009 and March 2014, 22 patients meeting the inclusion criteria were included (mean age 53 (SD 18) [range 19–88]; mean delay post-stroke at onset, 63 (29) [27–141] days; see detailed characteristics in Table 1). The four monthly clinical and robot-mediated evaluations occurred at the following mean delays post stroke: M2, 63 (29) [range 27–142] days (program onset); M3, 98 (32) [51–180] days; M4, 131 (28) [74–180] days; and M5, 167 (31) [120–249] days.

**Table 1:** Patient characteristics

Number	22
Age (years)	53 (18)
Gender	9 W
Side of hemiparesis	12 R
Time since stroke (days)	63 (29)
Etiology	I (15), H (7)
Duration of robotic training (days)	103 (13)

Data expressed as mean (SD). W, Women; R, Right; I, Ischemia; H, Hemorragia

### Clinical outcomes

**Fig. 2:**
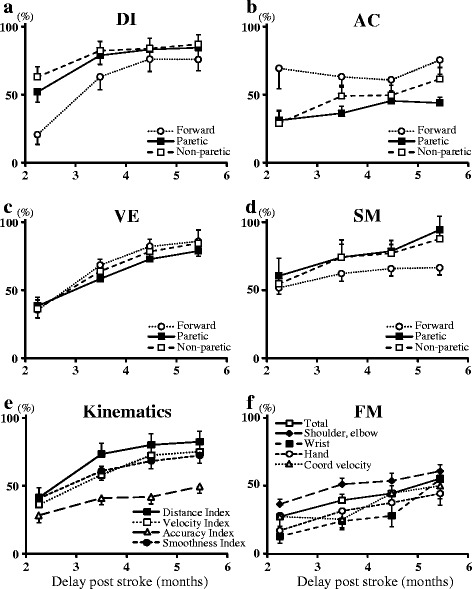
Kinematic and clinical changes over time. **a** DI: Distance index (%); **b** AC: Accuracy index (%); **c** VE: Velocity index (%); **d** SM: Smoothness index (%); **e** The four robot-based kinematics, all directions pooled, are represented; **f** FM: Fugl Meyer total score and sub-scores are represented. Coord velocity subscore, “Coordination velocity” (rapid alternating elbow movements). Results expressed as mean ± standard error of the mean. For the sake of figure clarity, asterisks indicating significance of changes since M2 have not been added in **a**, **b**, **c**, **d**, **e**, **f**; please refer to Tables 2 and 3 for statistical results

The number of movements achieved by the patients ranged from 353 to 1295 per session, with no suggestion of decrease in alertness throughout sessions. The FM score changes are summarized in Table 2 and Fig. 2f. From M2 to M5, the FM total score improved by a mean of 18.5 pts. over a total of 66 (main effect, *p* = 1.5E^−8^; M2 vs M5, *p* = 1.6E^−3^; M2 vs M3, +7.9 pts. (+12%), *p* = 1.2E^−3^; M3 to M5 + 10.6 pts. (+16%), *p* = 4.3E^−3^). For only the sample of subjects with no missing data across visits (*n* = 15), results were similar: main effect, 1.5E^−8^; M2 vs M5, *p* = 1.6E^−8^; M2 vs M3, +9.6 pts., *p* = 1.2E^−3^; M3 to M5 + 7.5 pts., *p* = 4.3E^−3^). The first movements to improve were the proximal shoulder/elbow movements, with an increase of 5.3 pts. in the FM corresponding subscore (+15% with respect to the maximal possible score of 36) from M2 to M3 (M3 vs M2, *p* = 3.4E^−3^). No significant changes were seen in the wrist and hand subscores during that period of time (+1.1 pts. (+11%) and +2 pts. (+14%) respectively). From M3 to M5, the wrist subscore significantly improved by 3.1 pts. (+31%, *p* = 4.0E^−2^) while changes in shoulder/elbow (+3.4 pts., +9.5%) or hand (+1.8, +13%) were not significant. However, interaction between time and proximal vs distal location (PXI vs DXI) of Fugl Meyer changes was not found to be significant (*p* = 0.24).

**Table 2:** Clinical outcomes

Fugl-Meyer	M2 (*n* = 19)	M3 (*n* = 18)	M4 (*n* = 17)	M5 (*n* = 15)	M2 vs M5 *p*
Overall (66)	18.0 (8.0)	25.9 (12.1)^a^	29.3 (14.5)^a^	36.5 (12.1)^a,b^	1.6E^−3^
Shoulder/Elbow (36)	13.1 (5.3)	18.4 (6.8)^a^	19.3 (7.8)^a^	21.8 (6.5)^a^	5.7E^−5^
Wrist (10)	1.3 (2.0)	2.4 (2.6)	2.8 (3.1)	5.5 (5.5)^a,b^	8.5E^−4^
Hand (14)	2.4 (2.7)	4.4 (3.7)	5.3 (4.7)^a^	6.2 (4.6)^a^	2.5E^−3^
Coord velocity (6)	1.6 (1.8)	1.5 (1.6)	2.7 (2.0)^b^	3.0 (1.6)^b^	ns

Results expressed as mean (SD). In the first column, total score and subscores are indicated with each corresponding maximal possible score in brackets. Sample sizes slightly decreased from M2 to M5 due to missing data. Coord velocity subscore, “Coordination velocity” (rapid alternating elbow movements). ^a^vs M2: *p* < 0.05; ^b^vs M3: *p* < 0.05

### Kinematic parameters

Kinematic results are summarized in Table 3 and Fig. 2a–e. Initially, velocity was 37.1% of normal over a covered distance of 41.7% of normal (Table 3). Four missing data at M5 were imputed using the M4 value. Over the study period, there were improvements in the distance index, velocity index, accuracy index and smoothness index (main effect, DI, *p* = 1.0E^−8^; VE, *p* = 1.0E^−9^; AC, *p* = 2.9E^−3^ and SM, *p* = 1.1E^−3^, Fig. 2e). Distance, velocity and smoothness indices improved first, increasing by 76%, 71% and 63% respectively at M3 (vs M2, DI, *p* = 1.9E^−5^; VE, *p* = 5.5E^−4^; SM, *p* = 4.6E^−2^). Comparatively, the accuracy index improved by 44% in the same M2 to M3 period and by 74% in the whole M2 to M5 period (AC, *p* = 1.5E^−3^). From M3 to M5, velocity index improved by 31% only (*p* = 1.5E^−2^). When smoothness was measured using the number of zero crossings in the acceleration profile, the pattern of changes was the same, with faster rate of change from M2 to M4 than from M4 to M5 (data not shown). A time*direction effect was found for distance and accuracy (Fig. 2a, b). At M2 and M3, the distance covered in the forward direction was shorter than in the paretic direction (*p* = 9.3E^−9^, *p* = 1.5E^−14^, respectively) and than in the non-paretic direction (*p* = 3.8E^−2^, *p* = 2.1E^−3^, respectively). At M2, accuracy was greater in the forward than in the paretic direction (*p* = 2.3E^−4^) and than in the non-paretic direction (*p* = 1.9E^−6^). At M3 and M5, accuracy in the forward direction was greater than in the paretic direction only (*p* = 5.6E^−4^, *p* = 5.0E^−4^, respectively).

**Table 3:** Robot-based outcomes

	M2 (*n* = 22)	M3 (n = 22)	M4 (n = 22)	M5 (n = 22)	M2 vs M5 *p*
DI	41.7(34.4)	73.5(37.6)^a^	80.3(38.2)^a^	82.6(35.5)^a^	7.1E^−8^
VE	37.1(28.5)	63.5(39.4)^a^	77.9(39.4)^a^	83.4(41.7)^a,b^	3.1E-^9^
AC	28.7(25.3)	41.3(22.9)	42.1(24.4)	49.8(23.9)^a^	1.5E^−3^
SM	41.0(24.5)	67.0(42.6)^a^	70.0(29.4)^a^	79.3(40.2)^a^	8.3E^−4^

Results (average over three directions) expressed as mean (SD). In first column, DI, distance index (% control); VE, velocity index (% control); AC, accuracy index (% control); SM, smoothness index (% control). ^a^vs M2: *p* < 0.05; ^b^vs M3: *p* < 0.05

## Discussion

The present open-label study quantified the clinical and kinematic changes following a 3-month rehabilitation program combining shoulder/elbow robot-assisted training and conventional occupational therapy for the upper limb in late subacute stroke, i.e. during the 3rd, 4th and 5th month after the event. The decrease in the motor impairment was associated with an improvement of all kinematic parameters assessed. Clinical improvements occurred proximally first, then distally while kinematic improvements in active range of motion, movement velocity and smoothness preceded those in accuracy.

### Study limitations

This was not a prospective controlled study and the subject number was small. The improvements observed could thus have been due to lesion-induced plasticity, i.e. “spontaneous” recovery at the late subacute phase since the study did not involve a control group without robot [2]. In addition, the number of participants dropped at each assessment. This however, did not seem to affect the overall findings as similar results were found when considering the sample of subjects with no missing data across assessment visits (*N* = 15), as indicated in Results. Yet, the present data represent rare observations confronting clinical assessment and robot-derived kinematic measures in late subacute stroke (3rd, 4th and 5th months post stroke; despite heterogeneity across patients in the exact delays after stroke for each evaluation), as opposed to few studies that reported about the high rate changes that occur within the first 3 months post stroke [25–27]. Additional potential limitations include that measures of performance as assessed by the robot used in the present study refer to planar point-to-point motion under the assumptions of the minimum-jerk model, which can be questioned [44]. Finally, body size of patients was not collected, which might otherwise have been computed in the calculation of the distance index.

### Magnitude of improvement from M2 to M5 post stroke

Between Week 1 and M3 post stroke, robot-based kinematic measures have yielded marked improvements both in trained and untrained movements and have shown, not only to be able to predict clinical measurements, in particular the Fugl-Meyer, being perhaps also to be more sensitive than clinical assessments in measuring recovery of patients [25–27]. From M2 to M5 following stroke, the time window explored here, the magnitude and pace of upper limb motor improvements observed in this study in association with the combined therapy program (robot + conventional care) seemed relatively high compared to other longitudinal reports in subacute stroke [29–31, 45]. For example, from M2 to M5 post stroke the present study reports an increase of 18 points in the Fugl Meyer score, vs less than 13 points in the first 6 months in a previous survey [29].

Many studies investigated the effects of conventional rehabilitation and/or non-intensive therapies on upper limb motor recovery in subacute stroke [29, 30, 46]. However, it is accepted that augmented rehabilitation programs using exercises at high intensity and focused on the repetition of numerous specific active movements, are effective on motor outcomes in subacute or even chronic patients [7, 11, 16, 47–52]; of note, two recent trials using semi-intensive programs (3 sessions a week) for short periods of time (8–10 weeks) produced negative results [53, 54]. In the first study, time per session was described without details regarding the number of movements achieved and the modalities used to perform movement. In the second study, the group with “high dose” training actually did not exceed 300 movements per session, in a chronic population. The intensity achieved by patients in the present study ranged between 353 and 1295 movements per robotic session only, 5 sessions a week for three full months; this number of movements per session did not include the conventional therapy, for which the literature reports around fifty movements performed in standard occupational therapy sessions [19, 55, 56]. The high intensity used in the present study could thus have contributed to the magnitude of improvement observed.

The pattern of improvement is consistent with other reports of FM score recovery, including with the proportional recovery model recently suggested [57, 58]; in particular, the present data suggest no plateauing of the progression of FM scores by M5–6 post stroke, when following the combined rehabilitation program used in the study. It might have been interesting to pursue this program for another six months, to verify whether progression would have slowed down, like in previous reports of FM changes over the first year post stroke [58].

### Improvement in specifically trained areas?

The evolution of the Fugl-Meyer subscores over time during the combined rehabilitation program including robot-assisted shoulder/elbow training in the present study suggested that motor improvement started proximally in the arm, earlier than distally (shoulder/elbow vs wrist). The notion of preferential improvement in specifically trained body parts has already been reported between upper and lower body parts [7]. Within one limb however, the literature is more controversial [32, 33]. Preferential proximal improvement had been suggested in some of the previous studies of robot-assisted therapy focusing on the repetition of proximal movements of the upper limb in the subacute phase of stroke, which showed “task-specific” motor improvements of the arm, with no or little improvement observed in non-trained joints [14, 15, 34, 36]. Yet, another trial reported non-site-specific motor improvements in the distal upper limb after a highly intensive robot-mediated training program using progressive resistance in chronic hemiparetic patients, which might suggest a proximal-to-distal pattern of improvement [35]. In the present study, conventional occupational therapy may also have contributed to the late distal improvements, without involving the hypothesis of a proximal-to-distal pattern of improvement.

### Respective improvements of the different kinematic parameters – Why might accuracy change more slowly than distance and velocity?

This study confirms improvements in all the kinematic parameters assessed after the combined rehabilitation program [59]. However, the refined information on the timing of motor recovery of the upper limb provided by kinematic assessments may give us insight into the motor recovery process [24–28] and contribute to the newly emerging field of computational neurorehabilitation, which aims at modeling plasticity to understand movement recovery in subjects with neurologic impairment [60]. In the present study, kinematic changes were characterized by an early increase in distance, velocity and smoothness of the target-approach movement as soon as one month after therapy onset, while accuracy (straightness) of movement improved only after 2 months of practice. The data confirms recent evidence that improvement in movement velocity during training in hemiparesis occurs rapidly and may even predict long term changes in movement velocity [61]. In such cases of subacute stroke-induced hemiparesis, it is not surprising to observe markedly faster and smoother reaching movements especially as “spontaneous” recovery (lesion-induced plasticity) and rehabilitation-related recovery (behavior-induced plasticity) are intertwined - and might even potentiate each other - in the first six months post stroke. The combination of these four kinematic measures thus seems sensitive enough to detect small changes on motor performance and comforts the idea of a training-induced motor learning process in which progress over time does not necessarily have to plateau out [61, 62].

The slower change in accuracy over time compared to the others kinematics is a compelling finding. First, this adaptative behavior might chronologically follow, and be explained by, improved smoothness i.e. the gradual decrease in the number of movement arrests, resulting in gradually reduced number of sub-movements and thus of new risks of error along the ideal trajectory [25, 26, 61]. These results may also fit the well-known speed-accuracy trade-off that governs voluntary movements (Fitts’ law), whereby it would be difficult to improve both parameters simultaneouly, including in stroke-induced hemiparesis [62, 63]. These findings may finally support the model that submovements may blend as a mechanism of recovery from stroke [25, 26, 64]. The reason for that may be that the primary sensorimotor networks, which directely generate movements, may recover functionality prior to cerebellar-frontal circuits, which are responsible for “automatic” accuracy controls [65]. Finally, the fact that speed and distance recovery precedes that of accuracy may serve as a didactic model for physical therapy schools, which for generations have privileged training movement accuracy before movement speed and amplitude, which may seem “unnatural” in the face of the present findings on recovery of movement in hemiparesis [66–68].

Regarding smoothness in particular, it should be noted that the number of zero-crossings in the velocity profile may not be the most sensitive smoothness metric, particularly in mild movement impairments [43]. However, this method seemed retrospectively justified in the present study in which initial velocity was 37% of normal, in a movement itself 42% shorter than normal (Table 3), indicating severe movement impairment at baseline in this population. In addition, the number of zero-crossings of velocity may not be the best means to estimate movement smoothness if it does not account for movement time [69]. The smoothness index calculated in the present study might have been increased by slower motion (increasing the chances of zero crossings), which also increases the difficulty of control (it is more difficult to move slowly while performing smoothly). In other words, it is not possible to exclude that some of the improvement of smoothness may have related to the improvements in velocity, which is an issue with metrics of smoothness except when normalizing by movement time, a normalization that was not performed here.

With respect to differential performance and changes according to the direction assessed (paretic, non paretic and forward), the present findings clearly indicate better performance in the paretic and non paretic than in the forward directions, particularly early in the evolution. This seems partly in contrast with more homogenous deficits observed in Kamper’s previous work [70], although for severely affected subjects preservation of sideward vs forward reaching was shown in that study as well [70].

### Usefulness of forearm-supported, assisted, point-to-point planar tasks in rehabilitation of the paretic arm?

The human arm normally self-supports its weight at the shoulder and moves along curved paths, smoothly from point to point (joints rotate, so curved motion requires less spatial control). In the tasks trained using the robot, the “ideal” trajectories were considered linear and accuracy was measured based on these ideal linear trajectories. It could be questioned whether such linear movements are the best training tasks since they may not correspond to physiological body kinematics and therefore may not represent the most helpful rehabilitation tasks with respect to task-oriented training. Additionally, one may wonder whether pointing tasks represent an optimal exercise to promote recovery, i.e. whether patients could have simply performed gradually better the tasks requested by the robot, without involving true functional recovery. The concomitant improvements in Fugl-Meyer may be partially reassuring in that respect; in addition, improvements in tasks not trained by the robot in subacute stroke have previously been demonstrated [26, 71]. Finally, smoothness improvements in a movement for which the arm is supported may not carry over to real life tasks, in which increased cocontraction of antagonists such as elbow or forearm flexors in a non-supported upper limb may come to disturb movements, while these cocontractions might be partially masked in the artificial situation of forearm support [72]. In fact, one study has reported that unassisted reaching exercises improve movement smoothness more than assisted training [73]. Repeated practice of a challenging movement can produce lasting physiological changes in motor neural networks, and in motor function. The functional usefulness of tasks that are “not” or “less” challenging for patients (i.e. assisted repetition of overlearned movements) should be compared with the training of more difficult tasks. Indeed, based on the assumptions of the minimum-jerk model, higher levels of central nervous system command are likely to specify the trajectory of the hand rather than the exact motions of the joints to perform the reaching movements [74, 75]. In other words, assisted pointing tasks could be more helpful to train the ability to *conceive* the kinematic parameters of the movement required (e.g. an appropriate rehabilitation of apraxia), than for improving movement *execution* in spastic paresis [76].

## Conclusions

During an intensive 3-month upper limb rehabilitation program combining robot-assisted shoulder-elbow training and conventional rehabilitation care initiated two months following stroke in patients with severe residual motor deficit, proximal before distal motor improvement was observed. In addition, active range of motion and velocity improved before movement accuracy. These findings suggest that a rehabilitation program with large amounts of daily repetitive active movements over a prolonged duration may stimulate brain plasticity, toward the specifically trained parts of the upper limb first. The study also suggests that behavior-induced brain plasticity is associated with active range of motion and velocity improvements (movement *quantity*) before movement accuracy (movement *quality*), a finding that might be worth considering when designing rehabilitation objectives and programs. Further prospective and controlled investigations in larger samples of subacute stroke patients should explore recovery by controlling the following factors: duration of the rehabilitation program, intensity of practice, modalities offered by robot (“assist-as-needed” or unassisted therapy), delay of onset of the therapy and stroke severity at baseline. Measures of actual functional abilities should also be added to the present outcomes.

## Abbreviations

AC: Accuracy Index
ANOVA: Analysis of variance
CT: Computerized Tomography
DI: Distance Index
DSI: Distal Index
FM: Fugl-Meyer Assessment
MRI: Magnetic Resonance Imaging
PXI: Proximal Index
SD: Standard Deviation
SM: Smoothness Index
VE: mean Velocity Index
